# The molecular mechanisms that determine different degrees of polyphagy in the *Bemisia tabaci* species complex

**DOI:** 10.1111/eva.13162

**Published:** 2020-11-20

**Authors:** Osnat Malka, Ester Feldmesser, Sharon van Brunschot, Diego Santos‐Garcia, Wen‐Hao Han, Susan Seal, John Colvin, Shai Morin

**Affiliations:** ^1^ Department of Entomology The Hebrew University of Jerusalem Rehovot Israel; ^2^ Department of Biological Services Weizmann Institute of Science Rehovot Israel; ^3^ Natural Resources Institute University of Greenwich Kent UK; ^4^ School of Biological Sciences the University of Queensland Brisbane Qld Australia; ^5^ Ministry of Agriculture Key Laboratory of Molecular Biology of Crop Pathogens and Insects Institute of Insect Sciences Zhejiang University Hangzhou China

**Keywords:** *Bemisia tabaci*, constitutive and plastic expression, host adaptation, insect–plant interactions, molecular mechanisms, polyphagy

## Abstract

The whitefly *Bemisia tabaci* is a closely related group of >35 cryptic species that feed on the phloem sap of a broad range of host plants. Species in the complex differ in their host‐range breadth, but the mechanisms involved remain poorly understood. We investigated, therefore, how six different *B. tabaci* species cope with the environmental unpredictability presented by a set of four common and novel host plants. Behavioral studies indicated large differences in performances on the four hosts and putative specialization of one of the species to cassava plants. Transcriptomic analyses revealed two main insights. First, a large set of genes involved in metabolism (>85%) showed differences in expression between the six species, and each species could be characterized by its own unique expression pattern of metabolic genes. However, within species, these genes were constitutively expressed, with a low level of environmental responsiveness (i.e., to host change). Second, within each species, sets of genes mainly associated with the super‐pathways “environmental information processing” and “organismal systems” responded to the host switching events. These included genes encoding for proteins involved in sugar homeostasis, signal transduction, membrane transport, and immune, endocrine, sensory and digestive responses. Our findings suggested that the six *B. tabaci* species can be divided into four performance/transcriptomic “Types” and that polyphagy can be achieved in multiple ways. However, polyphagy level is determined by the specific identity of the metabolic genes/pathways that are enriched and overexpressed in each species (the species' individual metabolic “tool kit”).

## 1 INTRODUCTION

Herbivorous insects exhibit a wide range of feeding‐strategy interactions with their plant hosts, with the classic main division distinguishing between generalist and specialist habits. Species that can feed and adapt to plants from a diversity of botanical families or broad dietary niches are considered to be generalists, while specialist species feed on one specific family of plant hosts and utilize a narrow dietary niche (Ali & Agrawal, 2012; Birnbaum & Abbot, 2020; Raubenheimer & Simpson, 1999; Simpson & Raubenheimer, 1995). This dichotomic division is largely considered to be a good starting point, but its simplicity is continuously challenged. For example, the discovery of new complexes of cryptic species in the aphid (Hemiptera: Aphididae) and Tachinid (Diptera: Tachinidae) groups (Loxdale & Harvey, 2016; Loxdale et al., 2011; Loxdale, et al., 2011) encouraged researchers to move beyond the traditional categories of generalists and specialists, placing herbivorous insects somewhere between the two extremes, with the generalist species being argued to be in many cases unrecognized complexes of more specialized species (Loxdale & Harvey, 2016; Charlery de la Masselière et al., 2017; Loxdale et al., 2019). Yet, generalism without any signal of division to host‐specialist groups was shown to be highly prevalent in some evolutionary lineages, being not only the “probable” but even the “inevitable” evolutionary outcome in those lineages (Clarke, 2017). As it is agreed that generalism is quite rare (Jaenike, 1990), its presence within certain lineages can be hypothesized to associate with a unique set of largely unknown and restricted biological attributes (mechanisms) that promote the evolution of generalist lineages (Clarke, 2017).

It is widely believed, for example, that generalists and specialists insect herbivores differ in the strategies they use to cope with plants' secondary defense compounds (Ali & Agrawal, 2012; Heidel‐Fischer & Vogel, 2015; Vogel et al., 2014b). Generalists that need to handle a wide range of such defenses are likely to rely on more generalized mechanisms such as avoidance (Cornell & Hawkins, 2003), or an arsenal of detoxification abilities that utilize detoxifying enzymes and xenobiotic transporters (Dermauw et al., 2018; Després et al., 2007). Specialists, on the other hand, mostly depend on effective sequestration, effectors secretion, or specific detoxification mechanisms to cope or manipulate specific defense compounds present in their plant hosts (Engler et al., 2000; Ratzka et al., 2002; Sasabe et al., 2004). Besides the challenge of handling a broad versus a narrow arsenal of plant defenses, species that are significantly apart on the generalism–specialism spectrum might also differ in their nutritional requirements. First, generalist is likely to experience greater heterogeneity in the contents and mixtures of nutrients found in their host plants than specialists (Raubenheimer & Simpson, 2003). Second, generalist and specialist can differ in the nutritional balancing of proteins and carbohydrates, the two main dietary nutrients acquired from plants (Behmer, 2009). When given a choice between protein‐ and carbohydrate‐biased foods, generalist insect species, such as the lepidopterans *Spodoptera exigua*, *Heliothis virescens* and *Helicoverpa zea* select protein biased diets (Chiluwal et al., 2020; Deans et al., 2017; Kwang et al., 2006; Merkx‐Jacques et al., 2008). In comparison, oligo‐ and monophagous species, such as *Manduca sexta*, *Heliothis subflexa,* and *Spodoptera. exempta*, select diets with equal protein:carbohydrate contents or slightly carbohydrate biased, likely reflecting the nutrient content of their evolved host plants (Kimura et al., 1987; Kwang et al., 2006; Lee et al., 2004; Thompson & Redak, 2005).

An additional layer of complexity comes from the feeding behavior and the anatomy of the feeding apparatus (Bernays, 1998), as both generalist and specialist insect herbivores evolved capabilities that allow chewing, piercing‐sucking, mining, and boring of plant tissues (Bernays, 1998). For example, the importance of plant‐defensive compounds in determining patterns of plant–insect interactions are well established in generalist/specialist chewing‐insect herbivores, but more controversial in sap‐feeders and pollinators (Ali & Agrawal, 2012; Dermauw et al., 2018; Kim & Jander, 2007; Stevenson et al., 2017). Also, the nutrient intake space of both generalist and specialist phloem‐feeding insects is very different from that described above, as these species need to cope with a poor diet that contains high levels of sugar with low levels of essential components such as amino acids (Douglas, 2006). Interestingly, phloem‐feeding insects show an intake target (the amount and balance of nutrients that when ingested, result in maximum performance) not in favor of proteins, but heavily biased (8:1, 600:75 mM) in favor of carbohydrates (Abisgold et al., 1994). Moreover, the high carbohydrate content of the phloem sap generates high osmotic pressure across the insect gut (Ashford et al., 2000). To reduce the osmotic pressure produced, phloem‐feeding insects such as aphids and whiteflies utilize several mechanisms. Both aphids and whiteflies synthesize glucose‐dominated oligosaccharides via a transglucosidase activity and excrete the oligosaccharides in the honeydew (Cristofoletti et al., 2003; Price et al., 2007). At high sucrose concentrations, aphids and whiteflies also reduce osmotic pressure in the gut by isomerizing sucrose to the disaccharide melezitose (Ashford et al., 2000) and trehalulose (Byrne & Miller, 1990), respectively. In addition, in order to compensate for their unbalanced diet and to obtain an optimal intake of energy and nutrients, phloem‐feeding insects established mutualistic interactions with intracellular bacterial symbionts which provide their herbivore hosts with the required nutrients lacking in their diet, mainly essential amino acid, co‐factors and vitamins (Hansen & Moran, 2014).

We focused here on the whitefly *Bemisia tabaci* (Gennadius) (Hemiptera: Aleyrodidae), a phloem‐feeding cryptic species complex, containing at least 35 distinct species assigned to ~11 major clades (Barbosa et al., 2014; Hu et al., 2018). Many species in the complex were shown to be flexible in their diet breadth and capable of acquiring new plants hosts to their diet repertoire (De Barro et al., 2011), and at the same time, to significantly differ in their documented host range (Malka et al., 2018). In a previous study, we selected six species within the complex, representing different phylogenetic groups and documented host ranges, from narrow (~9 plant families) to extreme (~50 plant families) polyphagy (Malka et al., 2018). We tested if differences in the species expression profiles of a small subset of detoxification genes (~300 genes) on common and novel hosts, were shaped more by their phylogenetic relationships or by their ability to utilize multiple hosts successfully. Our analyses indicated that *B. tabaci* species that differed in their ability to accept or utilize multiple plant hosts, also differed in their detoxification expression patterns. We also described a common detoxification “machinery” shared between the more generalist species (Malka et al., 2018).

In this study, we extended our analyses to the full transcriptomes of the same six *B. tabaci* species, in order to understand the high‐level functional differences between the selected species and to identify the molecular mechanisms that might explain the huge variability in host adaptation among the species in the complex. For that, we utilized 87 transcriptomes (six species fed on four plant hosts and an additional control of 10% sucrose‐only diet) and profiled the gene expression patterns of each species, identifying this way a suite of pathways that might be associated with the species level of polyphagy. Our analyses indicated that the majority of genes in each species (85%), were not affected by the insects' diet. Moreover, plastic responses seemed not to be adaptive and mildly contributed to the performance of only one species. Within the 85% constitutively expressed genes, each species displayed its own unique expression pattern of metabolic genes. These findings suggest that polyphagy can be achieved in multiple ways in *B. tabaci*, but its level is determined by the specific identity of the metabolic genes/pathways that are enriched and overexpressed in each species (the species' individual metabolic “tool kit”), together with the expression levels of non‐metabolic genes in response to novel host plants.

## 2 METHODS

### 2.1 *Bemisia tabaci* and host plant species

Six species of *B. tabaci* representing four different genetic groups (De Barro et al., 2011) were selected for analyses: SSA1‐SG3 (Sub‐Saharan Africa 1, sub‐group 3, collected in Tanzania in 2013/maintained on *Manihot esculenta*), ASIA II‐1 (Asia‐II genetic group, species 1, collected in Pakistan in 2013/maintained on *Gossypium hirsutum*), New‐World 2 (hence after NW2) (New‐World genetic group, species 2, collected in Brazil in 2013/maintained on *Solanum lycopersicum*), and MEAM1 (Middle East‐Asia Minor species 1), MED‐Q1 (Mediterranean Q species 1) and UGANDA‐MED‐ASL (Mediterranean non‐silverleafing sub‐group from Uganda) (Africa/Middle East/Asia minor genetic group, collected in Peru in 2012/maintained on *Gossypium hirsutum*; France in 2011/maintained on *Capsicum annuum* and Uganda in 2012/maintained on *Ipomoea batatas*, respectively). The identity of the six species was verified using their mtCOI DNA sequences (deposited in study accession number PRJEB21948). At least 2 months (~3–4 generations) before starting the experiments, ~500 founders from each of the six colonies were transferred to eggplant, to allow them to establish on a common baseline host plant. Colonies were reared under standard conditions of 28 ± 2°C, 60% humidity, and a 14:10‐h light:dark cycle. The selection of the experimental host plants was made based on our previously published literature survey and host reconstruction analyses (Malka et al., 2018), which identified common host plants, shared by many *B. tabaci* species, and non‐common novel host plants that are utilized by only few species. Based on this, four host plants were selected, eggplant, a common host (*Solanum melongena*, cv. Black Beauty, Solanaceae/ Solanales), and three novel host plants, also known to produce toxic phytotoxins (Malka et al., 2018): pepper (*Capsicum annuum*, cv. California Wonder; Solanaceae/Solanales), cassava (*Manihot esculenta*, cv. MCol22; Euphorbiaceae/Malpighiales) and kale (*Brassica oleracea*, var. *sabellica*, cv. Dwarf Green Curled; Brassicaceae/Brassicales). All experimental plants were grown in rearing rooms maintained at 28 ± 2°C, 60% humidity, and a14:10‐hr light:dark cycle.

### 2.2 Performance score

The reproductive success of each species on the four host plants was evaluated by scoring the *F*_1_/*F*_0_ ratio. Adult pairs (*n* = 20–50) of *B. tabaci* from the colony of each species on eggplants were transferred in triplicates to eggplant, pepper, kale, and cassava plants. The adult females were allowed to lay eggs for 2 weeks and were then removed from the plant. After 30–40 days, the performance score was calculated by estimation the ratio of the emerging *F*_1_ adults to the number of the transferred *F*_0_ adults_._ The scoring scale of *F*_1_/*F*_0_ was between 1 and 5: *F*_1_/*F*_0_ = 0 (1), *F*_1_/*F*_0_ < 0.1 (2), 0.1 ≤ *F*_1_/*F*_0_ ≤ 0.5 (3), 0.5 < *F*_1_/*F*_0_ < 1 (4), and *F*_1_/*F*_0_ ≥ 1 (5). (Table S1).

### 2.3 Gene expression analysis

Raw RNA‐Seq data for each species were obtained from SRP127757 (Malka et al., 2018). While the Malka et al. (2018) paper focused on comparing the expression levels of a small subset of 298 detoxification genes, this study conducted global analysis of the full transcriptome. Moreover, as detailed below, this study applied K‐means clustering and enrichment analysis in order to understand the essential differences in high‐level functions and utilities of the biological system (pathways) between the analyzed species, which could not be assessed with the small detoxification dataset. The reads obtained were subjected to quality control using the FASTQC software (http://www.bioinformatics.babraham.ac.uk/projects/fastqc/). For mapping and expression analysis, a reference backbone of 46,898 genes dataset, established for MED‐Q1, was used (Appendix S1). The dataset was manually curated to include 298 detoxification genes from six families (Malka et al., 2018). The reads were mapped and quantified using RSEM (RNA‐Seq by Expectation Maximization), v1.2.18. (Li & Dewey, 2011). RSEM uses an expectation maximization algorithm to deal with reads that are mapped to several locations by dividing the counts between the locations, and at the end, it counts each read only once. The transcript reference was first prepared (rsem‐prepare reference), followed by rsem‐calculate‐expression with the parameter Bowtie2 (Langmead & Salzberg, 2012). The percentage of mapped reads ranged from 41 to 78. Genes that did not have at least 10 reads in 4% of the samples were filtered out. The RSEM gene quantification for all the remaining genes was used as input for the DESeq2 R package, version 1.10.1. The gene counts were normalized using DESeq2 defaults, taking into account the mapped read numbers of the different samples.

We made two tests for assuring the quality of the reference backbone dataset we used. We compared it to the *B. tabaci* MEAM1 transcriptome in NCBI (ASM185493v1, file GCF_001854935.1_ASM185493v1_rna.fna) using blastn. From the 24,428 transcripts in NCBI, 21,840 were found in our backbone dataset, and from them, 19,716 had more than 95% identity. Moreover, there were 22,562 genes in our transcriptome that were not found in the NCBI transcriptome. We then mapped our reads to the NCBI *B. tabaci* MEAM1 genome and used the NCBI annotations to count the reads of all genes. The percentage of reads mapped to genes was very similar between the two datasets (Table S2). Taken together, these analyses confirmed the two datasets to be of similar quality. As detailed in Malka et al. (2018), we also performed two tests to show that DNA sequence differences between the six *B. tabaci* species did not bias our results due to differences in mapping efficiency, using the aforementioned subset of 298 detoxification genes. We produced one assembled transcriptome for each of the six analyzed species, using RNA‐Seq data from all the species' RNA samples. Next, we used a “blast reciprocal best hit” approach to check the identity of each gene in the species' transcriptome to its putative orthologous gene in reference backbone dataset. The mean identity for all six species was higher than 95%. In addition, arcsin‐square‐root transformed proportions of percent identities showed only low correlations with estimated DESeq2 values of the detoxification genes among all possible insect species and plant species combinations (Pearson's *r* ≤ 0.31). Finally, to assess the possibility of virus contamination, we checked the reads of 10 randomly chosen samples with different levels of read mapping for contamination using the KAIJU server (Menzel et al., 2016). This server performs taxonomic classification for metagenomics. The reference used to compare the reads was proGenomes that includes 20 million protein sequences from bacterial and archaeal genomes from the proGenomes database and 9,334 viral genomes from NCBI RefSeq. The run mode was greedy, allowing mismatches to be able to identify any similarity to viruses. The results indicated a very low level of contaminated reads: between 0.07% and 0.1% that were classified as viral, between 1% and 2% that were classified as bacteria and 0.1% that were classified as archaea (Table S3).

Differential expression analysis was performed using a full two‐factorial model (Appendix S2). Pairwise comparisons were performed between plants within species and between species within plants (details on differentially expressed genes for each comparison are provided in Tables S4 and S5). All pairwise comparisons were applied with the parameter "cooksCutoff = FALSE.” False discovery rate (FDR) was corrected for all the 30,012 genes that were not filtered out. The 95% log2‐converted fold‐change range (2.5%–97.5% quantiles) was −3.65 to 4.14 between species and −1 to 1.2 within species. For visualizations by principal component analysis (PCA) and hierarchical clustering, DESeq2 rld values were used. Rld stands for regularized log transformation of the count data to a log2 scale. PCA, hierarchical and K‐means clustering were performed using the PARTEK GENOMICS SUITE software, version 6.6 (v6.6; St. Louis, MO, 2014). The correlation method was applied to calculate the dispersion matrix of the PCA, and the eigenvectors were normalized. For the hierarchical clustering, Pearson's dissimilarity and complete linkage were applied.

K‐means clustering was applied to differentially expressed genes (fold change ≥ 2 and false discovery rate ≤ 0.05 between any two conditions). Clustering was calculated on standardized rld values using Euclidian distance. The Davies–Bouldin index was used to determine the optimal number of clusters that the transcriptomics data classified into. The number of clusters was chosen based on the minimal index value to ensure a good separation between clusters and cluster homogeneity. This minimal value was manually inspected to ensure that the minimal index values reflect the biological variability in the dataset.

### 2.4 Enrichment analysis

Kyoto Encyclopedia of Genes and Genomes (KEGG) annotations were obtained by submitting the sequence of each transcript to the KEGG Automatic Annotation Server (KAAS, https://www.genome.jp/kegg/kaas/) and receiving its KEGG Orthology (KO) number (Moriya et al., 2007). These were submitted to the KEGG Mapper—Reconstruct Pathway (http://www.genome.jp/kegg/tool/map_pathway.html) for identifying the pathways. KEGG gene counts per term and/or cluster were performed using in‐house Perl scripts. Enrichment *p* values and false discovery rates were calculated by applying the hypergeometric test in R. (Appendix S2).

## 3 RESULTS

### 3.1 Species performance on four different host plants

**Figure 1:**
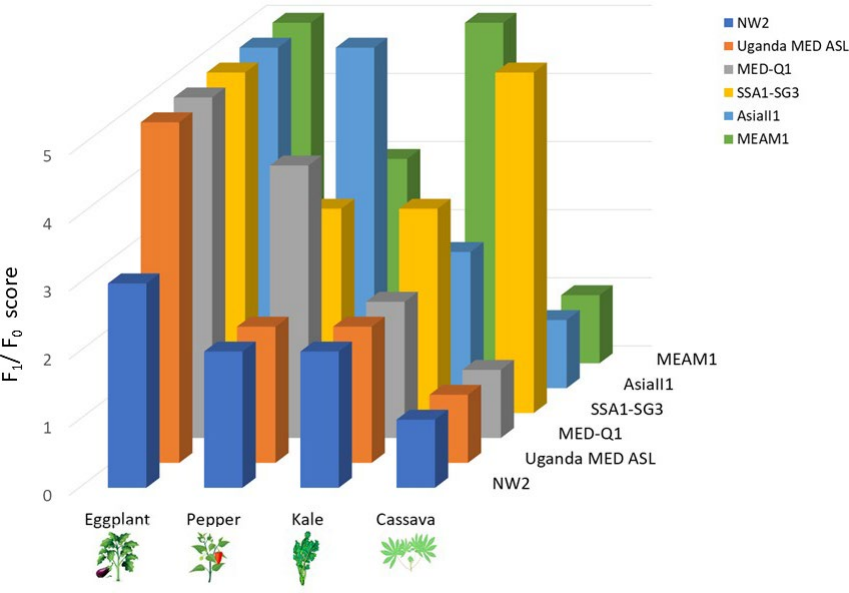
The estimated reproductive success of the six species on the four plant hosts. The F_1_/F_0_ scoring scale represents the percentage ratio between the number of emerging adults in the F_1_ generation and the number of initiating F_0_ adults that were transferred to the plants

We estimated the reproductive success (an ordinal score of the *F*_1_/*F*_0_ performance, see *materials & methods* for more details) of the six studied *B. tabaci* species (MED‐Q1, UGANDA‐MED‐ASL, NW2, SSA1‐SG3, MEAM1, and ASIA II‐1) on the four host plants (eggplant, kale, pepper, and cassava) (Figure 1, Table S1). The reproductive success score on eggplant was 3 for NW2 and 5 for all the other species. On kale, the reproductive success score was 5 for MEAM1, 3 for MED‐Q1, and 2 for all the other species. On pepper, the reproductive success score was 2 for UGANDA‐MED‐ASL and NW2, 3 for MEAM1 and SSA1‐SG3, 4 for MED‐Q1, and 5 for ASIA II‐1. On cassava, only the SSA1‐SG3 species produced viable progeny in the F_1_ generation with a performance score of 5 versus the other five species tested having a score of 1.

**Figure 2:**
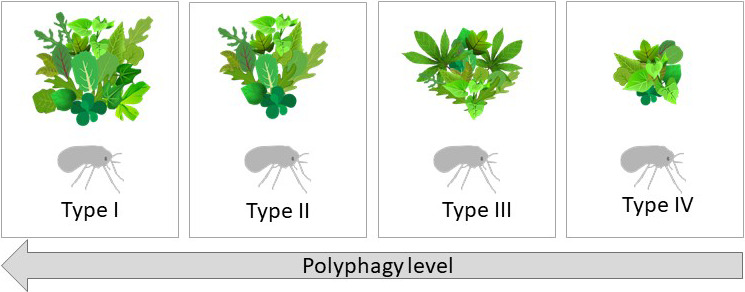
The size and variety of plant bouquets illustrate the “polyphagy level” of the four performance “Types” based on the reproductive success results presented in Figure 1. MEAM1 (“Type I”), MED‐Q1 & Asia II‐1 (“Type II”), SSA1‐SG3 (“Type III”), and Uganda MED‐ASL & NW2 (“Type IV”)

Based on these findings, the six species were divided into four performance types (Figure 2): “Type I,” generalist species with high reproductive capacity on all hosts excluding cassava (a plant species which seems to be outside the “normal” host range of the *B. tabaci* species complex). From the six species analyzed, only MEAM1 was considered a member of this type. “Type II,” generalist species showing an ability to utilize all host plants (excluding cassava), with reproductive success that can range from poor to good or very good. Both the MED‐Q1 and ASIA II‐1 species were considered members of this type. “Type III,” species with performance relatively similar to “Type II” species, but with unique specialization to a novel host. The SSA1‐SG3 species with its special adaptation to cassava was considered a member of this type. “Type IV,” species that are members of this type have the ability to utilize all host plants, as the three types above, but perform poorly on most of them. The NW‐2 and to a moderate extent the UGANDA‐MED‐ASL species were considered members of this type.

### 3.2 Patterns of gene expression of the six species on the different diets

**Figure 3:**
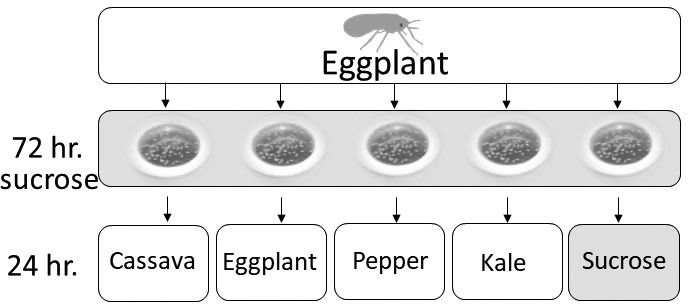
RNA‐Seq experimental setup. Two hundred newly emerged adults from each of the six species, grown on eggplant, were subjected to a feeding period of 72 hr on 10% sucrose diet, to obtain a standardized genes expression pattern. The sucrose diet lids were then replaced by clip cage lids, and the adults were transferred, for a feeding period of 24 hr, to the four experimental host plants. Control adults were subjected to additional 24 hr of feeding on 10% sucrose diet in new sucrose lids. Adults were then collected for RNA‐Seq analysis

**Figure 4:**
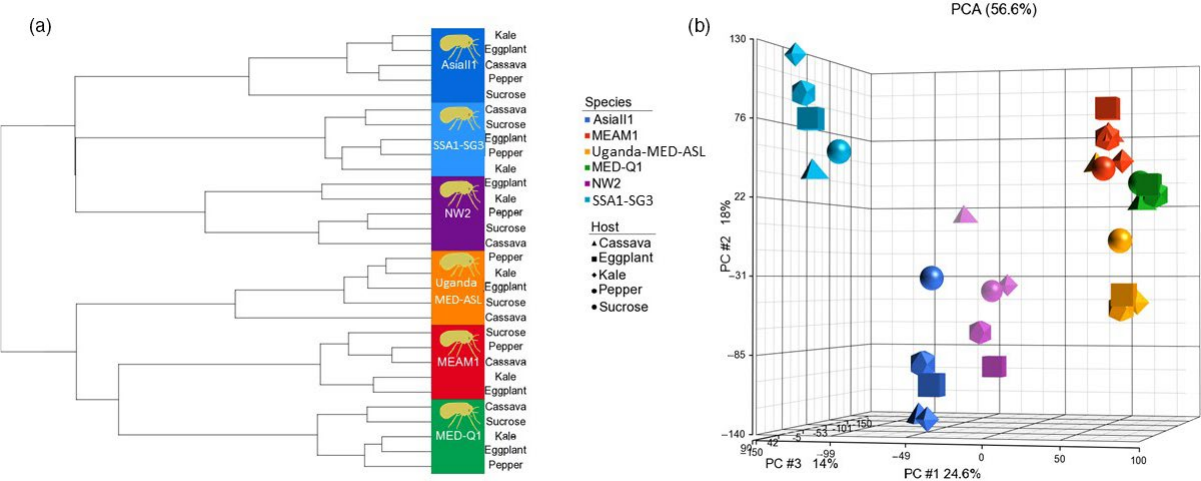
Exploratory analysis and samples' relationship. (a) Sample hierarchical clustering analysis was performed on the data from the all the expressed genes across the 30 insect‐species‐diet combinations (three biological replicates) with Pearson's dissimilarity distance and complete linkage methods. (b) Principal component analysis (PCA) was performed on the same data. The graph shows principal component 1 versus principal component 2 and 3 values for each combination (three biological replicates) in the study. The PCA and hierarchical clustering analyses were performed using Partek Genomics Suite software

**Figure 5:**
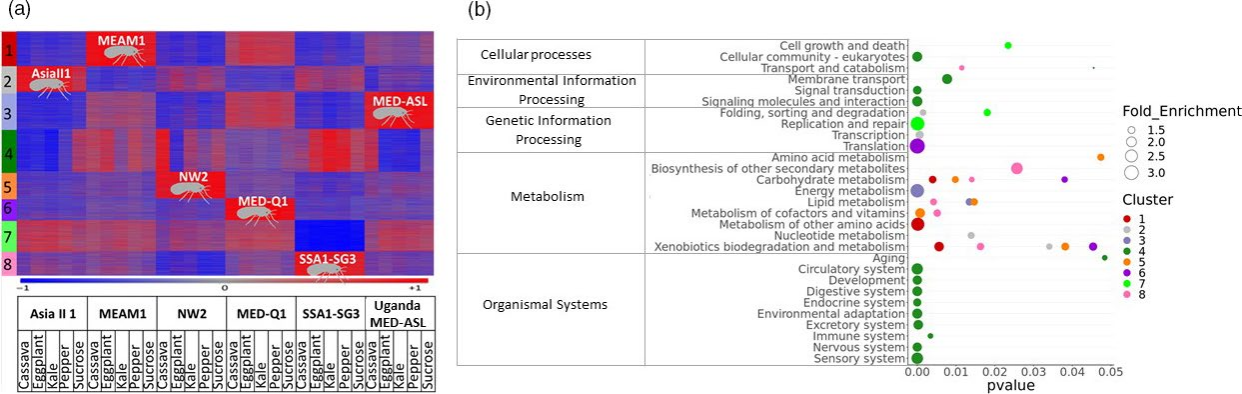
Gene expression profiles of six species of *B. tabaci*(ASIA II‐1, MEAM1, NW2, MED‐Q1, SSA1‐SG3, UGANDA‐MED‐ASL) on four host plants (cassava, eggplant, kale, pepper) and 10% sucrose diet. (a) K‐means clustering. Red represents upregulation, and blue represents downregulation (standardization was made on rld values for each gene across all 30 species and diet combinations). (b) Significantly enriched KEGG pathways (hypergeometric test, *p* ≤ .05) in the eight gene clusters displayed in figure a. Color‐cluster associations are the same as in figure a

We performed RNA‐Seq analyses on the six species after switching them from eggplant to a 24 hr feeding period on one of the four plant hosts or a 10% sucrose‐only artificial diet (Figure 3, Malka et al., 2018). Hierarchical clustering of the samples demonstrated that they group according to their level of phylogenetic relatedness and not according to their performance (“Types”): MEAM1, MED‐Q1, and Uganda‐MED‐ASL in one group, ASIA II‐1, SSA1‐SG3, and NW2, in the other (Figure 4a and Figure S1). In addition, no consistent grouping pattern of the five diets was found within each species. PCA analyses confirmed the associations determined by the hierarchical clustering (Figure 4b). Next, we identified specific gene expression patterns and groups of co‐regulated genes, by performing K‐means clustering on the list of 27,417 differentially expressed genes in at least one comparison. The list clustered into eight co‐expression groups (Figure 5a, Table S6), allowing the identification of groups that are uniquely upregulated mainly in one species and sometimes to some extent in additional one/s. Clusters 5, 6, and 8 were found to be uniquely overexpressed in the NW2, MED‐Q1, and the SSA1‐SG3 species, respectively. Genes in cluster 1 were overexpressed mainly in MEAM1 and to a certain level in MED‐Q1. Cluster 2 was mainly overexpressed in ASIA II‐1 and to some degree in NW2 and SSA1‐SG3. Cluster 3 was highly overexpressed in UGANDA‐MED‐ASL and moderately overexpressed in MED‐Q1. Cluster 7 grouped genes that were only downregulated in SSA1‐SG3. Interestingly, within‐species plastic expression related to the different diets (host plants or sucrose) was mostly found in cluster 4.

### 3.3 Functional analysis of enriched biological terms

Functional analysis of enriched biological terms was performed for each cluster using KEGG annotations (Figure 5b). Initial enrichment cutoff was set to *p* ≤ .05. Clusters 1, 2, 3, 5, 6, and 8 harbored genes involved in “metabolism,” which were specifically upregulated in one or more species (see above). Genes involved in “genetic information processing” were enriched mainly in clusters 2 and 7. Those involved in “cellular processes” were enriched in clusters 4 and 7. Cluster 4 was also enriched in genes involved in “organismal systems” and “environmental information processing.”

The KEGG database grouped the genes involved in “metabolism” into 12 functional “subcategories” or”super‐pathways” (https://www.genome.jp/kegg/pathway.html), including among others those responsible for the production/metabolism of amino acids, secondary metabolites and xenobiotics, carbohydrates, energy, lipids, nucleotides, glycans, and co‐factors/vitamins. Each “subcategory” contained several biochemical pathways, and 39 of them (Figure S2 and Table S7) were enriched in different clusters using a cutoff value of *p* ≤ .05. Moreover, some pathways were enriched in more than one cluster but with different gene sets. In some cases, genes in the same KEGG pathway were upregulated in one species and downregulated in another. The xenobiotic pathways: 00982, 00980, and 00983 were enriched in five clusters (Figure S2). Genes identified in these pathways mainly belonged to the UDP‐glucosyltransferases (UDPGT) and glutathione S‐transferases (GST) gene families and were expressed differently between species. For example, a specific set of *UDPGT* and *GST* genes in cluster 8 was upregulated in SSA1‐SG3 but downregulated in all other species. A different set of *UDPGT* and *GST* genes in cluster 6 was upregulated in MED‐Q1 and downregulated in all other species. The amino acid metabolic pathways were nearly unique in each cluster (00300, 00330, 00220, 00260, 00340, 00280, and 00290). The only exception was the 00250 pathway (“alanine, aspartate and glutamate metabolism”) which was enriched in two clusters. Genes that are involved in carbohydrates metabolisms were enriched in five different KEGG pathways: 00053 (“ascorbate and aldarate metabolism”) and 00040 (“pentose and glucuronate interconversions”) were enriched in clusters 5, 6, and 8; 00052 (“galactose metabolism”) was enriched in cluster 1 and 6; 00500 (“starch and sucrose metabolism”) was enriched only in cluster 1; and 00520 (“amino sugar and nucleotide sugar metabolism”) only in cluster 5. The genes identified in these pathways were mainly those coding for UTP‐glucose‐1‐phosphate uridylyltransferase, maltases and phosphatidylinositol and glycoside hydrolases. Other pathways that showed enrichment belonged to the “co‐factors and vitamins metabolism” and “lipid metabolism” super‐pathways. KEGG pathway 00860 (“porphyrin and chlorophyll metabolism”) was enriched in clusters 2, 5, 6, and 8, while 00830 (“retinol metabolism”) was enriched in cluster 5, 6, and 8. KEGG pathways 00565 (“ether lipid metabolism”) and 00100 (“steroid biosynthesis”) were enriched in cluster 8, 00561 (“glycerolipid metabolism”) was enriched in clusters 1 and 3, while 00140 (“steroid hormone biosynthesis”) was enriched in clusters 5, 6 and 8.

### 3.4 Correlation between performance and expression plasticity

As mentioned above, Cluster 4 was the only one to group genes responding to the different diets (host plants or sucrose). The plastic genes showed enrichment in KEGG pathways from the super‐pathways “organismal system”—“circulatory,” “development,” “digestive,” “endocrine,” “excretory,” “immune,” “nervous,” “sensory,” “aging,” and “environmental adaptation”; “environmental information processing”—“membrane transport,” “signal transduction,” and “signaling molecules and interaction”; and “cellular processes”—“cellular community (eukaryotes).” Spearman correlations between the mean expression levels of the enriched pathways in Cluster 4 and the reproductive success score of the species/“Types” on each of the four host plants, indicated the possible existence of two distinctive correlation patterns (Figure S3). “Type I” and “II” species, capable of performing well on multiple hosts, largely showed constitutive expression (very low level of plasticity), which did not correlate with the species performance on the different hosts (−0.10 ≤ Spearman's *r* ≤ 0.11, *p* ≥ .79, Figure S3a,b). In contrast, “Type III” and “Type IV” species largely showed a plastic response, which seemed to be adaptive only to some extent in “Type III” and non‐adaptive in “Type IV” (Figure S3c,d). However, these observations remain non‐conclusive for two reasons. First, the sample size was relatively small. Second, in the “Type III” species, the correlation between the mean expression level of the pathways and performance was not significant (Spearman's *r* = −0.89, *p* = .10). On the contrary, the mean expression level of the pathways was significantly negatively correlated with performance in both “Type IV” species (Spearman's *r* = −0.80, *p* = .016).

Following this, we tested the enrichment in Cluster 4, of eight gene families, known to play a role in the utilization of ingested sugars as a nutritional resource, the maintenance of osmotic balance and the detoxification of phytotoxins. Among these eight groups, only two, sugar transporters and ATP‐binding cassette transporters (ABC transporters) showed a significant enrichment (25 out of a total of 76 sugar transporters and 24 out of a total of 47 ABC transporters, Table S8). Again, there was clear difference in the expression pattern of the two gene groups between the outlined four performance “Types.” “Type I” and “II” species constitutively overexpressed (standardized rld values ≥ 1) ~10 sugar transporter and 1–2 ABC transporter genes (Table S9). The “Type III” species SSA1‐SG3 was similar to the “Type I” and “II” species by constitutively overexpressing high number of sugar and ABC transporter genes on three and four hosts (19 and 15, respectively), but differed from them by displaying also an inducible pattern of gene expression, mainly in the ABC transporters group (12 genes) in response to host switching to pepper and kale (Table S9). “Type IV” species were different from “Types I‐III” and constitutively overexpressed only two sugar transporters and no ABC transporter genes. Again, these species mainly displayed an inducible pattern of gene expression, as 19 and 13 sugar transporter and 18 and 15 ABC transporter genes were found to be overexpressed in NW‐2 and UGANDA‐MED‐ASL, respectively, in response to a specific plant host (mainly cassava) (Table S9).

### 3.5 The unique metabolic tool box of each specie/performance type

**Figure 6:**
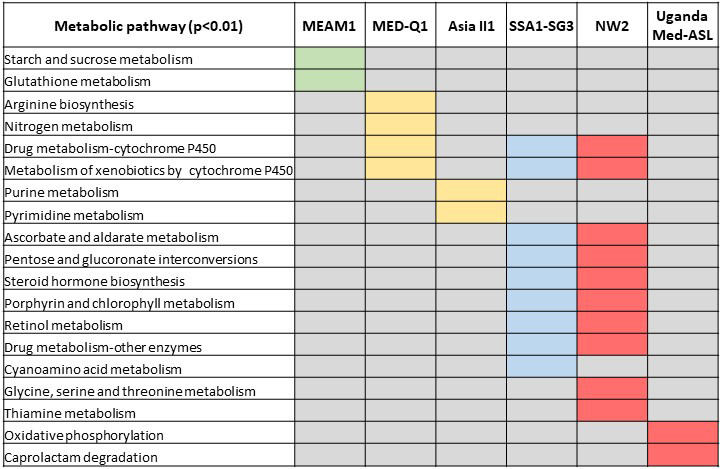
The unique metabolic “tool‐box” of each specie/performance “Type.” Significantly enriched KEGG pathways (hypergeometric test, *p* ≤ .01) in clusters associated with the “metabolism” category in Figure 5a. Green, yellow, blue, and red colors indicate enriched pathways in “Type I,” “Type II,” “Type III,” and “Type IV” performance groups, respectively

To identify the unique metabolic tool box of each species/performance “Type,” we focused on enriched pathways that showed a conservative cutoff of *p* ≤ .01. As indicated above, the phylogenetically (geographically) related species from the Mediterranean group presented similarity to some extent but it was clear that each species/“Type” has its unique metabolic tool box (Figure 6). The “starch and sucrose metabolism” pathway was found to be overexpressed and uniquely enriched only in the “Type I” species MEAM1, suggesting enhanced production of metabolites (glucose, fructose, trehalose, trehalulose, and oligosaccharides) that can be used in different metabolic pathways such as energy supply and storage but also in osmoregulation. MEAM1 also uniquely overexpressed genes involved in “glutathione metabolism.” Most of the overexpressed pathways, uniquely enriched in “Type II” species were found to be involved in amino acid biosynthesis and recycling of ammonia (“purine metabolism,” “pyrimidine metabolism,” “arginine biosynthesis,” and “nitrogen metabolism”). These pathways provide additional sources of energy production through purine and pyrimidine metabolism or the production of fumarate (intermediate in the citric acid cycle) during “arginine biosynthesis.” Species that are members of the “Type III” and “Type IV” groups, uniquely overexpressed gene clusters that were enriched in nine and ten pathways, respectively. Both “Types” showed enrichment in two carbohydrate metabolic pathways that are central to the conversion of glucose. The first, “ascorbate and aldarate metabolism” pathway forms UDP‐glucose, which is then oxidized to UDP‐glucuronic acid, and mainly used for the detoxification of toxic compounds through conjugation (Chen et al., 2019; Jiang et al., 2019). The second is the “pentose and glucuronate interconversions” pathway which generates NADPH and pentoses, mainly for maintaining/regenerating the cellular detoxifying and antioxidative defense systems (Agledal et al., 2010) and the synthesis of nucleotides and nucleic acids (Ceddia et al., 2003), respectively. The “Type III” species SSA1‐SG3, was the only one showing enrichment of overexpressed genes involved in the metabolism of cyano amino acids, amino acid derivatives that contain a cyanide group. As indicated above, SSA1‐SG3 showed specific adaptation to cassava plants that produce cyanogenic glucosides (Alves, 2002). “Type IV” species uniquely showed enrichment of overexpressed genes in the “glycine, serine and threonine metabolism” pathway, which are essential for energy homeostasis and amino acid metabolism through the citric acid cycle. This pathway was previously shown to be involved in cold and hypoxia tolerance in insects (Cui et al., 2017; Li et al., 2015). Species from “Types II, III and IV” showed enrichment of overexpressed genes in several pathways within the “xenobiotics biodegradation and metabolism” super‐pathway. These pathways are mostly involved in the detoxifying of phytotoxins and/or free radicals.

## 4 DISCUSSION

The development of a large set of genomic approaches in the last decade has enabled a remarkable growth in our understanding of the molecular mechanisms that facilitates host plant use by generalist and specialist herbivorous insects and how these mechanisms facilitate or constrain host shifts (Birnbaum & Abbot, 2020). Generalists are considered to harbor “general purpose genomes,” with variation that is persistently “tested by selection” on alternative plants. Expression plasticity may be abundant, and generally adaptive when challenged by new stressors (Birnbaum et al., 2017; Schweizer et al., 2017), facilitating broad niche occupancy. Specialists, on the other hand, may have “specialized genomes,” characterized by genomes that are “untested” in alternative environments (Huang et al., 2016). Most genes are expected to be constitutively expressed, and because the specialist can be considered “naïve” to new environments, initially non‐adaptive patterns of plastic expression is expected when new stressors are encountered (Birnbaum & Abbot, 2020).

It is quite clear, however, that these observations/predictions are likely to apply more to generalists chewing and mesophyll feeders that show large transcriptional changes during host switching (e.g., De La Paz Celorio‐Mancera et al., 2013; Dermauw et al., 2013; Govind et al., 2010; Grbić et al., 2011; Vogel et al., 2014a; Zhurov et al., 2014) or more specialized chewing insects that show relatively low level of plasticity when switched from their regular host to alternative hosts (Muller et al., 2017). In the latter study, the authors kept a population of the Brassicaceae specialized mustard leaf beetle, *Phaedon cochleariae*, for more than 40 generations on *Brassica rapa*, and then split it to three sub‐populations that continuously fed on *Brassica rapa*, *Nasturtium officinale,* or *Sinapis alba* for multiple generations. After 26 generations, gene expression comparisons between 10 days old larvae feeding on their latest host or one of the other host plants indicated very low (0.6%–2.8%) to low (7.1%) percentage of differentially expressed genes, mostly related to general cellular processes, metabolism, and digestion (Muller et al., 2017). On the other hand, a recent study compared larvae performance and gene expression changes in the generalist chewing herbivore *Spodoptera exigua* when feeding on three selected host plant species: cabbage, maize, tobacco, and a control artificial diet (Breeschoten et al., 2019). *Spodoptera exigua* larvae showed significantly higher performance when feeding on maize and artificial diet compared to cabbage and tobacco. Many of the 2,586 genes that were differentially expressed between the four diet treatments were found to be involved in immunity, digestion, detoxification, and cuticle formation and peritrophic matrix remodeling. Moreover, the authors detected a plant‐specific pattern of expression only when the larvae were fed on the two less‐suitable hosts (cabbage and tobacco), suggesting that polyphagy in *S. exigua* relies on a diverse and flexible set of genes that allow survival on a wide array of host plants (Breeschoten et al., 2019). Complementing findings were obtained using two additional *Spodoptera* species showing different degrees of polyphagy: *Spodoptera littoralis*, with a broad host range and two *Spodoptera frugiperda* strains primarily adapted to rice or maize (Roy et al., 2016). Performance assays using maize and a semi‐artificial diet indicated that the more polyphagous species, *S. littoralis* performed worse on maize than the two *S. frugiperda* grass‐adapted strains, while the performance of the three insect taxa was similar on the semi‐artificial diet. Comparative gene expression assays indicated that the greatest number of differentially expressed genes (26%) was found between the *S. littoralis* larvae fed on maize and the semi‐artificial diet, compared to 16% and 8% in the rice‐and maize‐adapted *S. frugiperda* strains, respectively. Similar to the results in *S. exigua*, the expression of genes involved in: digestion, metabolism, detoxification, transport, immunity and peritrophic matrix remodeling, was notably higher when feeding on maize (compared to the semi‐artificial diet), suggesting again that adaptive response of generalist chewing insects to a sub‐optimal plant diet mostly relies on the induction of large set of genes involved in these functions (Roy et al., 2016).

In contrast, the early transcriptomic responses (24 hr) to diet shifts of the six *B. tabaci* species analyzed here were mainly characterized by a constitutive “non‐changed” expression, with only a minority of genes showing overall plasticity even when the insects experienced a novel host plant like cassava. Moreover, the gene expression patterns obtained in cluster 4 (the only cluster showing significant within‐species plastic expression related to the different host plants) supported the above predictions only to a certain degree. “Type I” and “II” *B. tabaci* species, capable of performing well on multiple hosts, largely showed constitutive expression also in cluster 4, suggesting the presence of non‐plastic “general purpose genomes” that provide in most cases sufficient standing ability to utilize new hosts. In contrast, “Type III” and “Type IV” species largely showed a plastic response, which seemed to be adaptive only to some extent in “Type III” and non‐adaptive in “Type IV.” This raises the possibility that “Type IV” species harbor “more specialized” genomes that present general plasticity that was not tested by selection, making it in many cases non‐adaptive. Limited transcriptional responses, after transfer for 3 hr and 24 hr to a host plant, a non‐host plant or an artificial diet, were also reported in the generalist aphid *M. persicae* and the cereal aphid *Rhopalosiphum padi* (Thorpe et al., 2018). Taken together, these data suggest that phloem‐feeding insects might differ from other herbivorous insects feeding guilds in their early responses to new hosts. The main differences might be related to the different challenges faced upon host switching. As already indicated, the involvement of plant defense compounds in determining the fate of insect–plant interactions in generalist sap‐feeders are more questioned and controversial than in generalist chewers. For example, a wide variety of plant defense compounds are stored as glucoside conjugates. Only after the sugar is hydrolyzed by β‐glucosidase enzymes upon tissue damage, the released active aglucones are further modified to form substances that are toxic or deterrent to herbivores (Halkier & Gershenzon, 2006; Morant et al., 2008). Our knowledge of how sap‐feeding insects circumvent the activation or toxic effects of activated compounds is limited but it is generally believed that these insects do not damage the tissue extensively enough to cause the mixing of plant β‐glucosidases and glucosylated defense compounds and hence the formation of the active aglucones (Walling, 2008).

On the other hand, phloem‐feeding insects face a unique challenge when feeding on phloem sap that contains an abundance of simple sugars, few essential amino acids as well as other nutrients and relatively low concentration of plant secondary metabolites (Douglas, 2003). Therefore, it is not surprising that many of the metabolic pathways that were enriched and overexpressed in the analyzed *B. tabaci* species relate to the metabolism of carbohydrates and amino acids. Moreover, although the concentration of sugars and the osmotic pressure of the phloem sap varies among plants, the osmotic pressure is always higher in the phloem sap than in the phloem‐feeding insect (Douglas, 2003). As a result, it can be assumed that the mechanisms that phloem feeders are using for sugars homeostasis have an effect on their host plant acceptance. For example, it can be hypothesized that phloem‐feeding species that are able to achieve perfect homeostasis in sugar concentrations, independently of the external sugar concentrations that are present in a range of host plants, are the ones that should be considered as more generalist species. Our division of the *B. tabaci* species complex into four performance/gene expression “Types” supports this idea, as the number of constitutively overexpressed sugar transporters was 10, 10, and 19 in “Type I, II and III” species and only two in the “Type IV” species. Moreover, in the “Type IV” species (NW‐2 and MED‐ASL), the sugar homeostasis was more affected by the identity of the host plant (more than 90% of the sugar transporters were induced in response to the novel host plant compared to less than 50% in species belonging to “Types I, II and III”). Transporter‐mediated uptake of dietary sugar was previously shown to be essential in the biology of phloem‐feeding insects. For example, their silencing in the brown planthopper (BPH) *Nilaparvata lugens*, significantly interfered with the insects' longevity, reproduction, development, and viability (Ge et al., 2015; Kikuta et al., 2012). In addition, the *Acyrthosiphon pisum* genome was found to contain a relatively large number of genes encoding predicted sugar transporters, likely resulting from recent gene duplications that allow the efficient handling of high sugar concentrations in the insects' gut (Price et al., 2010). Support to the possible link between variation in sugar transporters expression and diet acceptance also comes from parallel experimental systems. A recent study on *Drosophila melanogaster*, which feeds on rotting fruits that contain sugar and yeast (Markow, 2015), brought evidence that whole body depletion of a sugar transporter highly expressed in the fly midgut, results in lethality after 3 days on a high sugar diet, and is accompanied by a characteristic starvation phenotype (Francis et al., 2020) The authors conclude that sugar transporters mediate their effects on starvation resistance by regulating glucose metabolism, raising the possibility that expression levels of sugar transporters might also be involved in determining if a plant will be considered as a host or non‐host (causing starvation) by phloem‐feeding species.

It appears that our division of the *B. tabaci* species complex into four performance/gene expression “Types” can be generalized and applied to other hemipteran systems, taking the well‐studied aphid superfamily as our main comparative model. For example, the aphid species *M. persicae*, which is globally distributed and presents a host range of >400 plant species (Blackman & Eastop, 2000), and *R. padi*, which is also distributed world‐wide and feeds on a wide range of grasses (Dixon, 1971), are likely to be aphid representatives of “Type I” and “Type II” species. As already indicated above, both species showed extremely limited transcriptional response (<10 genes) in the first 24 hr upon transfer to a host, non‐host plant, or artificial diet treatments (Thorpe et al., 2018) (Thorpe et al., 2018). The scale *Paratachardina pseudolobata* might be another good example of a hemipteran “Type I” generalist, as it has been recorded on more than 300 host plant species and was reported to be invasive widespread. Gene expression analysis of *P*. *pseudolobata* on three different host plants revealed that only 2.5% of the genes were differentially expressed across all comparisons. Moreover, of the 1,196 putative detoxification genes that were expressed, only 23 (1.9%) were significantly overexpressed on any one host (Christodoulides et al., 2017).

Species belonging to “Type III” show transcriptomic characteristics with similarities both to “Types I and II” and to “Type IV” species, and are unique in showing specialization to a novel and well‐defended host plant. Within the *B. tabaci* complex, the best example is the adaptation of species within the SSA group to cassava (Berry et al., 2004). Outside the *B. tabaci* complex, the best hemipteran example might be the adaptation of *M. persicae* to tobacco (*Nicotiana tabacum*) that led to a formation of a sub‐species, *M. persicae nicotianae* (Blackman, 1987). Adaptation to tobacco is driven by constitutive overexpression of the P450 monooxygenase gene *CYP6CY3*, which allows the tobacco‐adapted insects to efficiently detoxify nicotine (Bass et al., 2013). Recently, nicotine tolerance in *M. persicae nicotianae* was also associated with overexpression of *UDPGT* genes (Pan et al., 2019). It is important to note that both the cassava‐adapted species of *B. tabaci* and the tobacco‐adapted species of *M. persicae* can still utilize other plants as host, but seem to show reduced performance levels (Malka et al., 2018; Nikolakakis et al., 2003). One possible explanation to our findings that “Type III” species share transcriptomic and performance similarities with both “Types I and II” and “Type IV” species, is the possibility that “Type III” species are in a transformation phase of acquiring a novel host into their normal host range, a process which seems important/advantageous to the general fitness of the species (Nylin & Janz, 2009). As a result, these species are likely undergoing genetic accommodation and the acquisition of quantitative genetic changes that can either increase or decrease the expression level and environmental responsiveness of various gene families, which is likely to affect their performance on some hosts (Levis & Pfennig, 2016).

Species belonging to “Type IV” differ from the three “Types” above mainly by showing a much “weaker” generalist habit. They are still likely to be capable of utilizing many plants as hosts but perform poorly on most of them. The only *B. tabaci* species we analyzed that perfectly matched this description is NW‐2. UGANDA‐MED‐ASL showed many of the outlined characteristics, but seemed to be capable of performing well on some hosts, suggesting larger variation in the definition of this performance “Type” (Vyskočilová et al., 2019). In a broader sense, these two species might be the *B. tabaci* versions of host‐specialized species described mainly in the aphid lineage. For example, the cotton‐melon aphid, *Aphis gossypii*, might be considered as a “Type IV” species. It has a wide host range of hundreds of plant species belonging to various families such as Cucurbitaceae, Malvaceae, Solanaceae, Rutaceae, and Asteraceae (Blackman & Eastop, 2000), but many *A. gossypii* populations were found to form more specialized strains that utilize only a subset of host plant species in their recorded host range (Ma et al., 2019). Comparative analysis of gene expression of three populations of *A. gossypii*: cotton‐specialized, cucurbit‐specialized, and a cucurbit‐specialized reared on cowpea, revealed two very different expression patterns. Only 3,941 genes from 38,398 (10.3%) were differentially expressed between the cotton‐ and cucurbit‐specialized populations feeding on their home plant, mostly associated with sugar metabolism, immune, antioxidative and detoxification systems and salivary secretions. However, more than 15,000 genes (>40%) were found to be differentially expressed when these two populations were compared to the population feeding on the novel host cowpea. This time, the differentially expressed genes were mostly enriched in the KEGG super‐pathways “organismal systems” and “cellular processes,” indicating a significant stress‐response (Zhang et al., 2017). It is interesting to note that this response was quite similar in its characteristics to the non‐adaptive plastic response that “Type IV” *B. tabaci* species presented when switching to feed on a novel/non‐suitable host plants (Figure S3d), raising the possibility for the existence of an hemipteran common “stress‐response tool‐box.”

In conclusion, analysis of 87 transcriptomes belonging to six species of *B. tabaci* feeding on four plant hosts and a sucrose‐only artificial diet, clearly indicated that many of the enriched and overexpressed pathways that differ between the species relate to the transport and metabolism of sugars and the synthesis of a range of amino acids, and less to pathways involved in the detoxification of phytotoxins. Still, our current knowledge is quite limited. Many more comparative studies, integrating transcriptomic and fitness metrics in different hemipteran systems are required, before any general micro‐ and/or macro‐evolutionary argument can be made on the evolution of diet breadth in generalist and specialist phloem‐feeding species. Primary metabolism constrains and osmoregulation/dehydration challenges might play a significant role in the guild interaction with its host plant. We encourage future research to focus on the interface between primary and secondary metabolism as a promising essential tool for achieving better understanding of the regulatory networks that controls carbon flux in phloem‐feeding insects, likely uncovering the lineage unique diversification processes.

## AUTHOR CONTRIBUTIONS

Osnat Malka, Ester Feldmesser, Susan Seal, John Colvin, and Shai Morin conceptualized the study. Osnat Malka, Sharon van Brunschot, Ester Feldmesser, Susan Seal, John Colvin, and Shai Morin provided insects, genetic materials, and facilities. Shai Morin and John Colvin involved in funding acquisition. Osnat Malka, Ester Feldmesser, Sharon van Brunschot, Diego Santos‐Garcia, and Shai Morin involved in data acquisition and formal analysis. Osnat Malka, John Colvin, and Shai Morin developed the methodology. Wen‐Hao Han involved in construction and characterization of a cDNA library. Osnat Malka and Shai Morin drafted the manuscript. Ester Feldmesser, Sharon van Brunschot, Susan Seal, and John Colvin revised the manuscript.

## Supporting information

Table S1‐S3, S6‐S9

Table S4

Table S5

Fig S1

Fig S2

Fig S3

Appendix S1

Appendix S2

## Data Availability

Mitochondrial cytochrome oxidase I (mtCOI) DNA sequences of the six species and transcriptome sequence reads of the 72 samples will be deposited in the European Nucleotide Archive under the study accession number PRJEB21948.
