# Supplementary figures and images for: The molecular mechanisms that determine different degrees of polyphagy in the Bemisia tabaci species complex
Source: Evol Appl. 2020 Nov 20;14(3):807–20. doi: 10.1111/eva.13162 (PMC7980310; doi:10.1111/eva.13162)

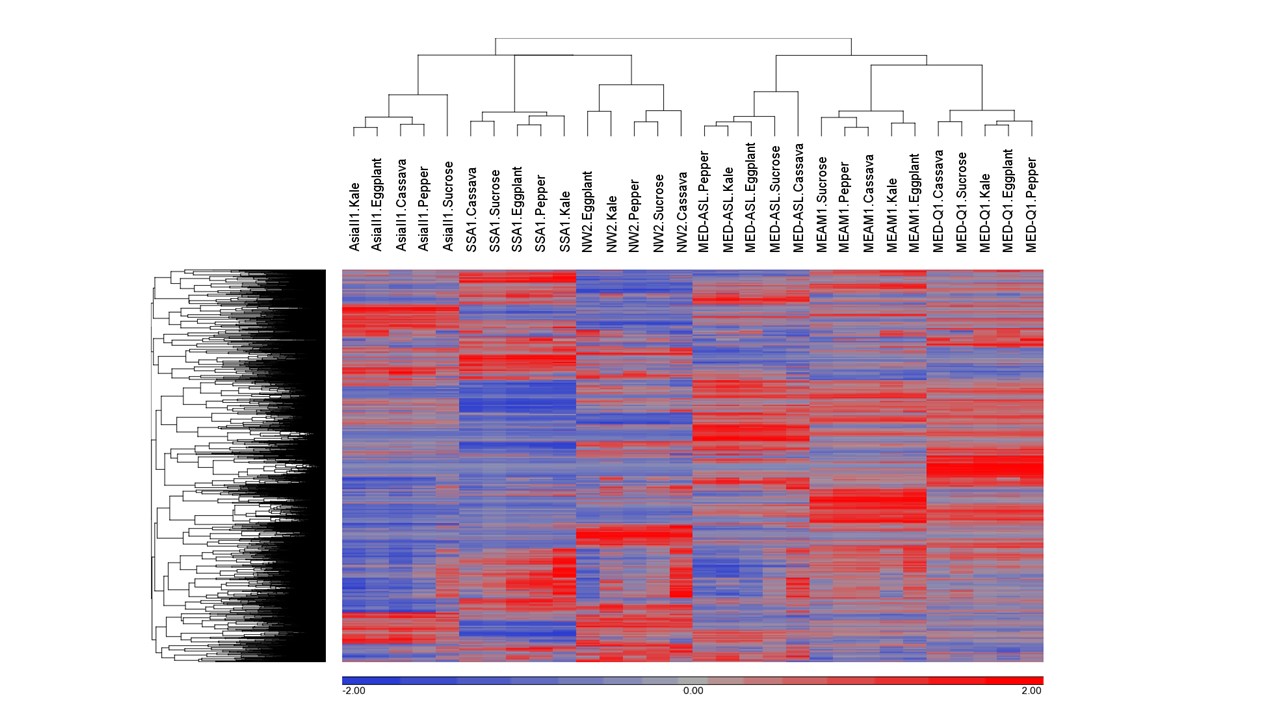

Supplement: Supplementary file 4 — Fig S1 [file EVA-14-807-s006.jpg]

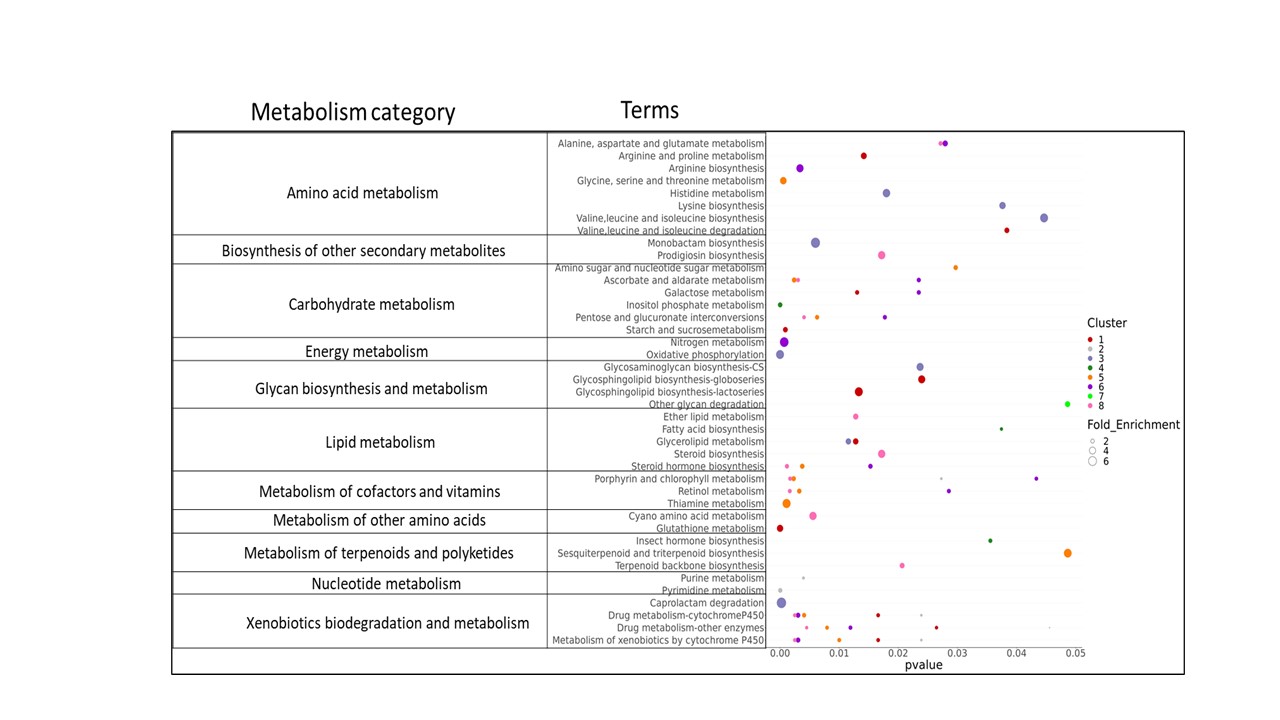

Supplement: Supplementary file 5 — Fig S2 [file EVA-14-807-s008.jpg]

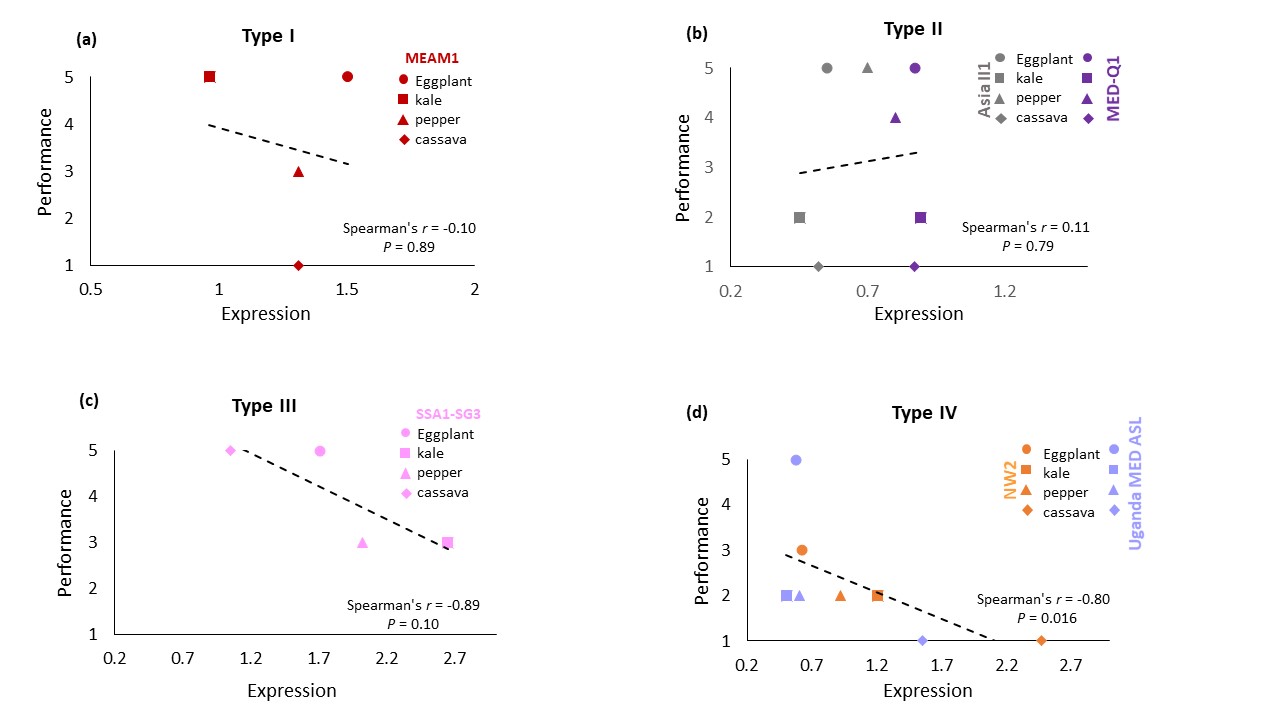

Supplement: Supplementary file 6 — Fig S3 [file EVA-14-807-s005.jpg]
