# Supplementary material for: The molecular mechanisms that determine different degrees of polyphagy in the Bemisia tabaci species complex
Source: Evol Appl. 2020 Nov 20;14(3):807–20. doi: 10.1111/eva.13162 (PMC7980310; doi:10.1111/eva.13162)
Supplement: Supplementary file 8 — Appendix S2 [file EVA-14-807-s003.docx]

**Coding scripts:**

**deseq2 between plants (for table S4)**

library("DESeq2")

setwd("T:/projects/wis/esterf/shai/DiffExp_all/deseq2_noAsia1")

samples <- read.delim("info_no.txt", header=FALSE)

countData <- read.delim("filtered_matrix_no_Asia1.txt")

rownames( countData ) <- countData$Gene

countData <- countData[ , -1 ]

countData =round(countData)

species = samples[,2]

plants = samples[,3]

batch = samples[,5]

ExpDesign <- data.frame(row.names=colnames(countData), species = species, plants=plants, batch =batch)

dds = DESeqDataSetFromMatrix(countData, colData=ExpDesign, design= ~plants +species + species:plants)

dds1 <- DESeq(dds)

# estimating size factors

# estimating dispersions

# gene-wise dispersion estimates

# mean-dispersion relationship

# final dispersion estimates

# fitting model and testing

#8 rows did not converge in beta, labelled in mcols(object)$betaConv. Use larger maxit argument with nbinomWaldTest

res1 <- results(dds1,cooksCutoff=FALSE)

dat1 = as.data.frame(res1)

de<-array(NA,c(1,4))

for (k in 1:6){

dds.sp<- dds[, colData(dds1)$species == levels(species)[k]]

as.data.frame(colData(dds.sp)[,"plants"])

design(dds.sp) <- ~ plants

dds.sp <- DESeq(dds.sp)

resultsNames(dds.sp)

for (i in 1:4){

j=i+1

for (j in j:5){

label = paste(levels(species)[k],"-",levels(plants)[i], "_", levels(plants)[j], sep="")

res <-results(dds.sp, cooksCutoff= FALSE,contrast=c("plants",levels(plants)[i],levels(plants)[j]))

res.m=as.data.frame(res)

colnames(res.m) = paste (label,colnames(res.m),sep=".")

dat1<-cbind(dat1,(res.m))

bb<-(abs(res.m[,2])>=1 & res.m[,6]<=0.05 & is.na(res.m[,6])==FALSE)

Names<-dimnames(res.m)[[1]][bb==TRUE]

l<-length(Names)

de<-rbind(de,cbind(Names,rep(levels(species)[k],l),rep(levels(plants)[i],l),rep(levels(plants)[j],l)))

}

}

}

de<-de[-1,]

write.table(dat1,"DE_full_between_plants_sucrose_Agust2017.txt", sep="\t")

write.table(de,"list_DE_full_between_plants_sucrose_Agust2017.txt", sep="\t")

**deseq2 between species (for table S5)**

library("DESeq2")

setwd("T:/projects/wis/esterf/shai/DiffExp_all/deseq2_noAsia1")

samples <- read.delim("info_no.txt", header=FALSE)

countData <- read.delim("filtered_matrix_no_Asia1.txt")

rownames( countData ) <- countData$Gene

countData <- countData[ , -1 ]

countData =round(countData)

species = samples[,2]

plants = samples[,3]

batch = samples[,5]

ExpDesign <- data.frame(row.names=colnames(countData), species = species, plants=plants, batch =batch)

dds = DESeqDataSetFromMatrix(countData, colData=ExpDesign, design= ~plants +species + species:plants)

dds1 <- DESeq(dds)

# estimating size factors

# estimating dispersions

# gene-wise dispersion estimates

# mean-dispersion relationship

# final dispersion estimates

# fitting model and testing

#8 rows did not converge in beta, labelled in mcols(object)$betaConv. Use larger maxit argument with nbinomWaldTest

res1 <- results(dds1,cooksCutoff=FALSE)

dat2 = as.data.frame(res1)

de2<-array(NA,c(1,4))

for (k in 1:5){

dds.pl<- dds[, colData(dds1)$plants == levels(plants)[k]]

as.data.frame(colData(dds.pl)[,"species"])

design(dds.pl) <- ~ species

dds.pl <- DESeq(dds.pl)

for (i in 1:5){

j=i+1

for (j in j:6){

label = paste(levels(plants)[k],"-",levels(species)[i], "_", levels(species)[j], sep="")

print(label)

res <-results(dds.pl, cooksCutoff= FALSE,contrast=c("species",levels(species)[i],levels(species)[j]))

res.m=as.data.frame(res)

colnames(res.m) = paste (label,colnames(res.m),sep=".")

dat2<-cbind(dat2,(res.m))

bb<-(abs(res.m[,2])>=1 & res.m[,6]<=0.05 & is.na(res.m[,6])==FALSE)

Names<-dimnames(res.m)[[1]][bb==TRUE]

l<-length(Names)

de2<-rbind(de2,cbind(Names,rep(levels(plants)[k],l),rep(levels(species)[i],l),rep(levels(species)[j],l)))

}

}

}

de2<-de2[-1,]

write.table(dat2,"DE_full_inside_plants_sucrose_July2017.txt", sep="\t")

write.table(de2,"list_DE_full_inside_plants_sucrose_July2017.txt", sep="\t")

**functional_counts (used for table S6)**

#!/usr/local/bin/perl

use strict;

# Ester Feldmesser 22.2.18

#v4

# The script count number of genes that belong to a functional category in predifined groups of genes (clusters, up or down regulated genes).

#the groups file should include the cluster or name of the group of genes and the gene name, tab separated.

#the function file (tab separated) includes the gene name in the first column and then functional categories that can be comma or tab separated.

#input files

my $groups = $ARGV[0];

my $function= $ARGV[1];

#output file

my $out =$ARGV[2];

my $out_names = $ARGV[3];

my $info= $ARGV[4];

if (($#ARGV != 4)or($ARGV[0] eq "-h" )) {

print "Usage: \n";

exit;

}

#global variables

my %idInGroup =();

my %count =();

my %group_count=();

my %names = ();

my %function_count=();

my $sum=0;

open (RESULT, ">$out") || die "cannot open \"$out\": $!";

open (RESULT2, ">$out_names") || die "cannot open \"$out_names\": $!";

open (INFO, ">$info") || die "cannot open \"$info\": $!";

open (IN, $groups) || die "cannot open \"$groups\": $!";

my $tt = <IN>;

while (my $line = <IN>) {

chomp($line);

my ($group,$id)= split (/\t/, $line);

$idInGroup{$id} =$group;

#print "$group,$id\n"; print "$idInGroup{$id}\n";

}

open (FF, $function) || die "cannot open \"$function\": $!";

while (my $line = <FF>) {

chomp($line);

my ($id,@annot)= split (/\t/, $line); #kog

# my ($id,@more)= split (/\t/, $line); #kegg

# my @annot= split (/,/, $more[0]);

$group_count{$idInGroup{$id}}++; #counts the number of annotated genes per group

#print "a $id\tb $idInGroup{$id}\tc $group_count{$idInGroup{$id}}\n";

my $i=0;

my (@unique)=();

$unique[0] = $annot[0];

foreach my $item(@annot) {

unless($item eq $unique[$i]) {

push(@unique,$item);

$i++;

}

}

#print "@unique\n";

foreach my $item (@unique){

if ($item=~/\w/){

$function_count{$item}++;

if (exists $idInGroup{$id} ){

my $ref= $idInGroup{$id}."-".$item;

$count{$ref}++;

$names{$ref}= $id .",".$names{$ref};

}

}

}

}

foreach my $key ( keys %group_count ) {

print INFO "$key\t$group_count{$key}\n";

$sum = $sum + $group_count{$key};

}

foreach my $key ( keys %function_count ) {

print INFO "$key\t$function_count{$key}\n";

}

print RESULT "cluster\tcat\tcount.cat.cl\tcount.cluster\tpop\tcount.cat\n";

foreach my $family ( keys %count ) {

my($gr,$cat)=$family =~/(.*?)-(.*)/;

#print "$gr, F: $function_count{$gr}\n";

print RESULT "$gr\t$cat\t$count{$family}\t$function_count{$gr}$group_count{$gr}\t$sum\t$function_count{$cat}\n";

print RESULT2 "$family\t$names{$family}\n";

}

close (IN);

close (RESULT);

close (RESULT2);

close (FF);

**KEGG pathways categories (for table S7)**

#!/usr/local/bin/perl

use strict;

# Ester Feldmesser 5.11.18

# The script reformats kegg Pathway Reconstruction Result to the format needed in functional_counts.v4.pl

#input

my $function= $ARGV[0];

#output file

my $function_reformated =$ARGV[1];

if (($#ARGV != 1)or($ARGV[0] eq "-h" )) {

print "Usage: perl reformat_kegg.pl kegg_KO.file reformated_kegg.file\n";

exit;

}

#global variables

my %function_code=();

my %annotation;

my $code;

open (REF, ">$function_reformated") || die "cannot open \"$function_reformated\": $!";

open (FF, $function) || die "cannot open \"$function\": $!";

while (my $line = <FF>) {

chomp($line);

my @genes;

$line =~ s/\s\s+//; #remove spaces at the begining

$line =~ s/"//;

# print "$line\n";

if ($line =~ /^\d{5}.*/) {

$line =~ /(\d+)(.*)\(/;

$code=$1;

#print "$1,$2\n";

$function_code{$code} = $2;

#print "SPL $line\t$1\t$2\n";

}elsif (($line !~/K.*/)&($line =~ /\d/)) {

#my (undef,$genes)= split('\t',$line);

#print "elsif: $line\n\n";

$line=~ s/\s\s+//g;

if ($line =~ /,/){

@genes =split(',',$line);

}else{

$genes[0] =$line;

}

foreach my $i (@genes){

$i =~ s/ //g;

if (exists $annotation{$i}){

$annotation{$i} = join("\t",$annotation{$i},$code);

}else { $annotation{$i} = $code; }

}

}

}

foreach my $key (keys %annotation){

print REF "$key\t$annotation{$key}\n";

}

foreach my $key (keys %function_code){

print "$key\t$function_code{$key}\n";

}
